# Medical Clinic

**Published:** 1883-03

**Authors:** Alfred L. Loomis

**Affiliations:** Professor of Pathology and the Practice of Medicine in the Medical Department of the University of the City of New York, held in Bellevue Hospital


For Contents See Page 192.

THE

### Independent Practitioner.

Vol. IV. March, 1883. No. 3.

### Original Communications.

We are not responsible for the opinions expressed by contributors.

Article I.

### MEDICAL CLINIC.

BY ALFRED L. LOOMIS, A.M., M.D.,

Professor of Pathology and the Practice of Medicine in the Medical Department of the University of the City of New York, held in Bellevue Hospital.

CASE I.

*Gentlemen* :—This man, æt. 34, has reached the 10th day of his illness. Until this attack he had been perfectly healthy. His sickness began with a "chilly sensation"—not a distinct chill—accompanied by headache of moderate severity, pains in the back, nausea and vomiting of what he calls "greenish water." Epistaxis occurred but once.

Two days after painless diarrhœa came on, and each discharge made him "feel more comfortable." He had from ten to twelve discharges in the twenty-four hours. Anorexia, emaciation and a steady loss of strength have been marked, yet he worked for eight days after the occurrence of the first symptom, *i.e.*, the "*chilly sensation.*" The patient states he has not been intemperate during the eight months preceding his illness. For a month past he has worked in a damp basement; but there was no one ill in the house in which he lived.

When admitted to my wards yesterday he had a temperature of 103° Fahr. Ten grains of quin. sulph. were administered last evening, and to-day his temperature fell to 101° Fahr. His pulse is now ninety-six and his temperature 100½° Fahr. His tongue is red, with moist edges, and a dark brown stripe through its centre. His mind is clear; and there are no symptoms indicating marked disturbance of the nervous system. His abdomen is slightly tympanitic; but there is no gurgling in the right iliac fossa. The spleen and liver are both enlarged.

We notice an abundant eruption on the abdomen, purplish in color, somewhat elevated, disappearing only upon firm pressure.

His eyes are not suffused and the countenance has a natural expression. An urinary examination gave only negative results.

A physical exploration of the chest fails to reveal any abnormality.

Now, gentlemen, is there any symptom, or group of symptoms that can lead directly to a diagnosis? Typhoid fever suggests itself. The appearance of the tongue does not settle the question, for such a condition of the tongue may be present independent of typhoid fever. The same may be said of the diarrhœa. The fact that there has been epistaxis does not warrant a diagnosis of typhoid fever.

The eruption is too abundant, too dark, and does not disappear quickly enough on pressure for a typhoid eruption. It rather resembles the eruption of *typhus fever*, without, however, the mottled appearance of the latter.

His temperature is not the typical typhoid temperature. At the time of his admission it was 103°, and after a single dose of quinine it fell to and remained at 100° Fahr. I have never seen the temperature in typhoid fever yield so readily and so long to a single moderate dose of quinine. Moreover, the diarrhœa has ceased without treatment. Typhoid diarrhœa usually continues much longer, unless it is controlled. He has not the characteristic typhoid countenance.

We have, then, a patient who has a continued fever without a single characteristic symptom of typhoid fever. We are not warranted in making the diagnosis of typhoid fever, unless there is present the characteristic "step-ladder" temperature, the "rose rash," the peculiar typhoid expression of the face and the tenderness and gurgling in the right iliac fossa, accompanied by the characteristic diarrhœa.

This man was the first to be sick among those who worked and lived with him.

To have typhoid fever there must be a specific typhoid poison, and there will, therefore, be a history of exposure to this specific contagion.

In the majority of cases of so-called *typhoid fever* occurring in this city, no evidence of specific infection can be traced.

Typhoid fever is transmitted from the sick to the healthy through the stools, but there is no evidence of such an exposure in this case.

There are, however, septic poisons entering our dwellings from many sources, which, combined with malarial poison, give rise to fevers which have many of the phenomena of typhoid fever, but they cannot strictly be classed under that head. I have called this "Typho-malarial Fever." It is not a good name, but it is better than the unclassified fevers of many authors.

In typhoid fever, when the specific element of the fever is once introduced into the body, the individual so infected must pass through the classical phases of the fever.

Much can be done in the way of modifying the symptoms, but when once the fever is developed it must run its course. Such is not the case in the type of fevers to which I am referring. The differential diagnosis between these fevers is often difficult at first, but the absence of the three characteristic symptoms of typhoid fever settles the question.

The question arises, may not the diagnosis be completed by a microscopical examination of the blood for the purpose of determining whether or not pigment is present? We certainly find pigment in the blood in well-marked cases of malarial fever. But I answer, not always.

What I wish to direct special attention to in connection with this case is the care one should take in drawing the line between these two forms of fever. One is a specific disease; the other is not.

One depends upon a specific poison, and requires the greatest possible care to prevent its spread; while the other is a disease which has no specific element.

[To be continued.]

---

THERE recently died in the Rochus-Spital at Buda-Pest, so says the *Wiener Med. Wochenschrift*, a woman æt. 70, in whom no spleen was found. Prof. Schenthauer made the autopsy, which showed that the spleen had not been absorbed from disease, but that it had never existed. It would have been interesting to know what was the condition of the lymphatics and medulla of the bones in this case.

